# Characterization of Heat Conduction Performance in Sodium Polyacrylate Hydrogels with Varying Water Content

**DOI:** 10.3390/ma19030454

**Published:** 2026-01-23

**Authors:** Nan Wu, Cuiying Fan, Guoshuai Qin, Xu Zhang, Zengtao Chen, Minghao Zhao, Chunsheng Lu

**Affiliations:** 1 School of Mechanics and Safety Engineering, Zhengzhou University, Zhengzhou 450001, China; wunan_2026@163.com (N.W.); xuzhang@zzu.edu.cn (X.Z.); memhzhao@zzu.edu.cn (M.Z.); 2 School of Electromechanical Engineering, Henan University of Technology, Zhengzhou 450001, China; gsqin@haut.edu.cn; 3 Department of Mechanical Engineering, University of Alberta, Edmonton, AB T6G 2E1, Canada; zengtao@ualberta.ca; 4 School of Civil and Mechanical Engineering, Curtin University, Perth, WA 6845, Australia

**Keywords:** sodium polyacrylate hydrogel, specific heat capacity, thermal conductivity, thermal relaxation, water content

## Abstract

Sodium polyacrylate (PAAS) hydrogel is a functional polymer known for its excellent water absorption, retention, and thermal stability; however, its thermal conductivity behavior in engineering applications remains insufficiently understood. In this paper, two experimental setups were designed and constructed to measure the specific heat capacity and thermal conductivity of PAAS hydrogel in liquid, powder, and fluid–structure coupled states. The results show that the thermal conductivity initially increases rapidly with increasing water content and then decreases, achieving a maximum enhancement of 66% compared with PAAS powder. In contrast, the specific heat capacity exhibits an exponential increase and asymptotically approaches that of water. These findings demonstrate the thermal properties of PAAS hydrogel can be effectively tuned by adjusting its water content. Based on a composite material parameter model, simple predictive relationships for both specific heat capacity and thermal conductivity were established as functions of water content. Numerical simulations using the Fourier heat conduction equation validate the proposed models, with thermal relaxation behaviors in good agreement with experimental observations. Therefore, this work not only quantifies the thermal conductivity performance of PAAS hydrogels but also provides practical predictive models for the thermal design of hydrogel-based materials with enhanced heat transfer efficiency in engineering applications.

## 1. Introduction

Hydrogels are cross-linked polymeric networks that retain large amounts of water within the spaces between polymer chains. Since their initial synthesis [1], they have evolved into diverse forms, typically prepared using water as a solvent. Hydrogels exhibit unique properties such as strong adhesion [2], thermal stability [3], and mechanical toughness [4], making them promising candidates for heat dissipation and cooling applications in medicine, agriculture, industry, and electronic information engineering [5,6,7,8]. Among these properties, thermal conductivity is particularly critical, as it directly governs performance. Understanding the heat transport behavior of hydrogels is therefore essential for clarifying heat transfer mechanisms, identifying key influencing factors, and designing advanced hydrogel structures with tailored thermal properties.

The thermal conductivity of hydrogels depends on multiple factors, including composition, structure, temperature, and water content. Most existing research has focused on tuning polymeric composites through the incorporation of additives such as metal powder, ceramics, and graphene [9,10,11]. For example, Yazdan et al. [12] reported that increasing the agarose fraction reduced conductivity, whereas doping with boron nitride significantly enhanced it. Water content also plays a decisive role in polyacrylamide hydrogels. Conductivity can increase by nearly 40% when water content rises from 23% to 88% by weight [13,14].

Molecular dynamics (MD) simulations have become a powerful tool for probing the microscopic mechanisms underlying hydrogel thermal properties. Using statistical thermodynamics, MD enables accurate predictions of thermal conductivity in polymer systems [15,16,17]. Luo et al. [18], for instance, identified the upper and lower bounds of polydimethylsiloxane conductivity by analyzing thermal transport in chains of different lengths. Similarly, Basem et al. [19] studies on polyacrylamide hydrogels loaded with cefazolin revealed that thermal conductivity remained nearly constant at 0.57 W·m^−1^·K^−1^. Xiao et al. [20] showed that conductivity of polymers increases more than fourfold as polymer pyrolysis progresses from 0% to 100%, while Xu et al. [21] found a non-monotonic dependence on water content. Conductivity rose with water content up to 85%, but decreased beyond this threshold, approaching the conductivity of pure water. These findings underscore the effectiveness of MD simulations for linking hydrogel microstructure to macroscopic thermal behavior.

Despite the insights provided by MD simulations, experimental verification remains essential for practical applications. Tél et al. [22] measured the thermal diffusivity of N-isopropylacrylamide hydrogels by exploiting their temperature-sensitive phase transition. Wu et al. [23,24] proposed a microscopic statistical model connecting anisotropic with deformation, predicting hydrogel behavior under tensile loading. Tang et al. [14] further demonstrated experimentally that cross-linking polyacrylamide with N-methylenebisacrylamide improved thermal conductivity by 54%. However, experimental investigations of thermal transport in hydrogels remain scarce compared with MD simulations.

Moreover, widely used techniques for measuring thermal properties, such as the transient plane source method [25], differential scanning calorimetry [26], and laser flash analysis, face significant limitations when applied to hydrogels. First, hydrogels with varying water contents are prone to surface adhesion, which can lead to probe contamination and poor interfacial contact. These issues accelerate probe wear and increase long-term maintenance and testing costs, thereby limiting the feasibility of rapid screening or repeated measurements on large numbers of samples. Second, these techniques often rely on measurements from a limited number of specimens, resulting in increased data dispersion and reduced statistical reliability. Consequently, to accurately and reliably characterize the thermal behavior of hydrogels, it is necessary to develop or adapt tailored experimental methods that enable high-throughput, low-cost measurements while maintaining sufficient accuracy. These are essential in supporting scalable hydrogel development and effective quality control.

Sodium polyacrylate (PAAS), a water-soluble polyelectrolyte hydrogel, is particularly attractive due to its excellent water absorption and retention capability. Its thermal relaxation characteristics are critical for evaluating responsiveness under dynamic thermal conditions. However, there are few reports on the thermal transfer properties of PAAS hydrogels, particularly dynamic thermal responses such as thermal relaxation, remain scarce, if not entirely absent. Moreover, the influence of water content on fundamental properties, including specific heat capacity and thermal relaxation behavior, is still poorly understood, which significantly limits the broader industrial application of PAAS hydrogels. Therefore, comprehensive experimental characterization of the thermal conductivity behavior and dynamic thermal response for PAAS hydrogels over a wide range of water contents is essential to advance their practical implementation. In this paper, to address this gap, we present an experimental study on the effect of water content on the thermal properties of PAAS hydrogel. Furthermore, we obtain predictive relationships to evaluate their heat transfer capacity. These efforts aim to provide both an experimental foundation and a predictive framework to guide the engineering and industrial application of PAAS hydrogels.

## 2. Thermal Conductivity Model

All chemical reagents, including refined acrylic acid, sodium hydroxide, N,N′-methylenebisacrylamide, and ammonium persulfate, were purchased from Shanghai Aladdin Biochemical Technology Co., Ltd. (Shanghai, China). The PAAS resin particles used in this study were synthesized as follows: 4 g of refined acrylic and 1 g of sodium hydroxide were dissolved in deionized water and stirred thoroughly. Subsequently, 0.07 g of N,N′-methylenebisacrylamide was added to ensure complete dissolution. The solution was degassed using an ultrasonic cleaner to remove bubbles, and 0.020 g of ammonium persulfate was introduced at room temperature as an initiator. Following the standard post-processing procedure [27], resin particles with a size distribution of 40–100 mesh were obtained.

**Figure 1:**
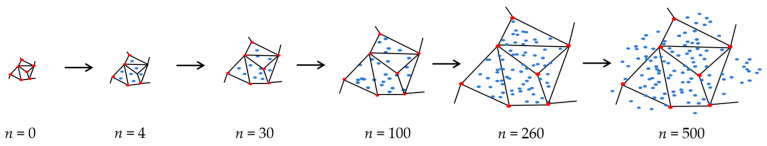
Schematic diagram of PAAS hydrogels with varying water contents. Red dots and black lines are polymer network; Blue dots are water molecules.

PAAS hydrogels are well known for their exceptional water absorption capability, being able to absorb several hundred times their own weight in water. The swelling behavior of the polymer network at different water contents is illustrated in Figure 1, where the dots represent the corresponding water content. Here, hydrogels were prepared using a swelling method, where water content was precisely controlled by adjusting the resin mass during hydration. The water content (*n*) is defined as the mass ratio of absorbed water to polymer resin [28], expressed as

(1) $$n=\frac{m_{w}}{m_{p}},$$

where *m_w_* is the mass of water and *m_p_* is the mass of the PAAS powder.

For samples with *n* < 100, thorough stirring was sufficient to achieve uniform swelling. For samples with *n* > 100, the PAAS powder was first immersed in water for 4.5 h, followed by sieving through a 120-mesh sieve for 0.5 h to ensure accurate water content. More than 260, water was found to have overflowed, as shown in Figure 1. Therefore, the saturated water content of PAAS hydrogel was determined as *n* = 260. Repeated measurements indicate that for *n* ≥ 4, the density deviation from that of water is less than 5%. In this regime, the hydrogel density (*ρ*) remains nearly constant with increasing water content and closely approaches the density of water (1 g·cm^−3^). As the present study primarily focuses on highly hydrated hydrogels, the analysis is therefore restricted to the range *n* ≥ 4. Within this range, density serves only as a constant proportional coefficient in the governing equations and does not significantly influence the observed thermal behavior.

Meanwhile, throughout the experiments, the heating time was limited to within 10 h and the temperature was maintained below 90 °C. Under these conditions, the overall mass change in the samples was negligible (<1%). Consequently, all measurements in this paper were conducted under ambient pressure and at temperatures no more than 90 °C.

### 2.1. Experimental Setup

**Figure 2:**
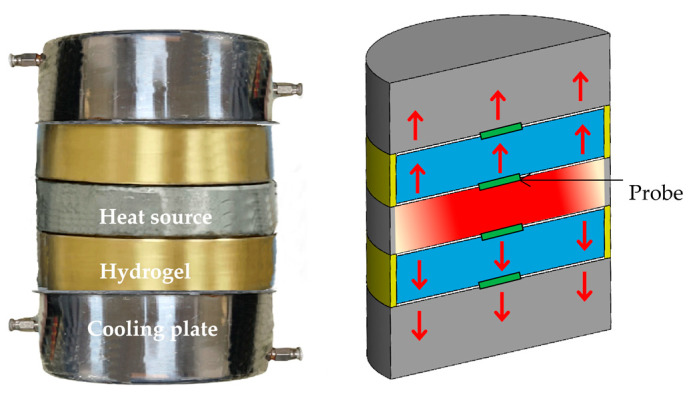
Schematic of the experimental setup used for thermal conductivity measurements. Red arrows indicate the direction of heat flow.

The thermal conductivity of PAAS hydrogels was measured following Fourier’s law of heat conduction and ISO 8302:1991 [29,30]. The experimental setup is designed and constructed. As illustrated in Figure 2, a heat source simultaneously heated two identical hydrogel samples placed symmetrically on either side. The apparatus comprised two cooling plates, two copper containers, a central heating source, and four thermocouples. Each copper container had a diameter of 10 cm and a thickness of 2 cm, while the cooling plates measured 10 cm in diameter and 5 cm in thickness. The hydrogels were placed inside copper containers to prevent flow and maximize contact with the heating plate. To minimize convective and lateral heat losses, both the sample and the central heating source were wrapped in thermally insulating material and tested in a still air environment. In addition, the entire setup was enclosed within an insulated chamber to further reduce heat exchange with the surroundings.

According to Fourier’s law, heat is transferred opposite to the temperature gradient. Based on the input power and measured temperature difference, the thermal conductivity *k_n_* of hydrogels with different water contents is calculated by

(2) $$k_{n}=\frac{P_{1}\;l}{2A_{0}\left(T_{1}-T_{0}\right)},$$

where *P*_1_ is the steady-state power of the heating plate, *l* is the hydrogel thickness, *A*_0_ is the contact area between the hydrogel and heating plate, *T*_0_ is the cooling plate temperature, and *T*_1_ is the steady-state temperature at the heating plate-hydrogel interface. In this experimental setup, the ratio between the steady-state power of the heating plate *P*_1_ and the corresponding temperature difference (*T*_1_ − *T*_0_) in the testing process changes with the variation in hydrogel with different water content.

For validation, Table 1 compares the results from this experimental setup with measurements obtained using the transient plane source method [25]. Here, *k_w_*, *k_p_*, and *k*_30_ represent the thermal conductivities of water, PAAS powder, and PAAS hydrogel with *n* = 30, respectively. The custom experimental setup features a larger sampling volume and reduced sensitivity to surface adhesion force, thereby mitigating the influence of local inhomogeneities on the measurement results. As a consequence, the relative standard deviation (RSD) is approximately 3%, indicating improved measurement consistency. Table 1 presents the averaged results obtained from five independent tests.

In contrast, measurements using the transient plane source method typically employ probes with diameters of 5–10 mm and require intimate contact between the sample and the sensor. Under these conditions, the RSD across different sampling points increases to approximately 7%, reflecting a higher degree of data dispersion. The corresponding averaged results, based on ten independent measurements, are also summarized in Table 1.

In the measurement of thermal conductivity for PAAS hydrogel, the results obtained using the custom experimental setup are in good agreement with those from the transient plane source method. The discrepancy between the two approaches is within 3.70%, confirming that the accuracy of the proposed setup satisfies the required measurement standards. In addition, the custom setup demonstrates improved stability and reliability. When probe cost and long-term maintenance requirements are also considered, this setup offers clear advantages for characterizing the thermal behavior of hydrogels and other soft materials. Consequently, it is well suited for measuring the thermal conductivity of PAAS hydrogels over a wide range of water contents.

### 2.2. Experimental Results

**Figure 3:**
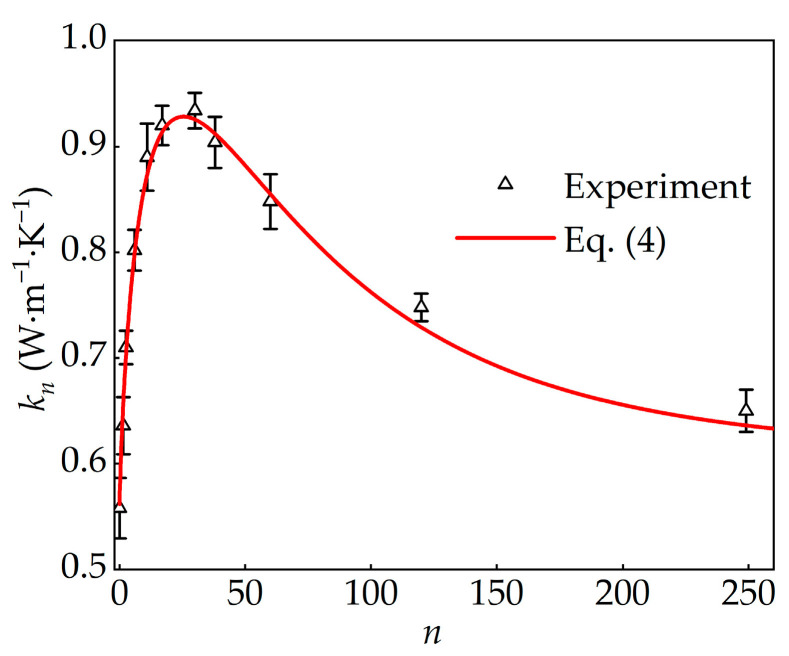
Thermal conductivity of PAAS hydrogels under different water contents.

At room temperature (20 °C), five independent experiments were performed for each condition, and the results are shown in Figure 3. The data indicate that the thermal conductivity of PAAS hydrogels is strongly dependent on water content during the swelling process. Specifically, the thermal conductivity initially increases with increasing water content and then decreases as water content continues to rise. This nonlinear behavior is consistent with previous observations for polyacrylamide hydrogels [21].

This trend can be attributed to the evolution of the hydrogel network structure during swelling, as illustrated in Figure 1. At low to moderate water contents, network expansion facilitates more efficient energy transfer pathways, thereby promoting heat conduction and raising thermal conductivity [31]. However, once swelling exceeds a critical level, the polymer network becomes overstretched and increasingly tense, which impedes efficient energy transport. As a result, the thermal conductivity gradually decreases and ultimately approaches that of water at high hydration levels.

These results highlight the ability of PAAS hydrogel to tune its thermal properties through water content. Obviously, the maximum conductivity of 0.93 W·m^−1^·K^−1^ was observed at *n* = 30, corresponding to a 66% increase compared with PAAS powder.

### 2.3. Prediction Model

Previous studies have established theoretical models based on MD simulations to describe the relationship between hydrogel thermal conductivity and water content [21]. Although effective, these models are often too complex for practical engineering use. To simplify the description while retaining accuracy, an exponential decay function was employed to characterize the nonlinear dependence of thermal conductivity on water content [32]. The model is expressed as

(3) $$k_{n}=k_{w}+\left(k_{p}-k_{w}\right)\left(1-a_{1}n\right)\exp(-{\left(a_{2}n\right)}^{a_{3}}),$$

where *a_i_* (*i* = 1, 2, 3) are dimensionless constants determined from experimental data. Using the experiment results in Figure 3, a fitting curve with a correlation coefficient of 0.992 was obtained, yielding

(4) $$k_{n}=k_{w}+\left(k_{p}-k_{w}\right)\left(1-1.55n\right)\exp\left(-{\left(0.11n\right)}^{0.57}\right).$$

Figure 3 presents the experimental measurements together with the fitted Equation (4), illustrating the quality of the fit across the full range of *n*. Obviously, this model accurately captures the nonlinear variation in PAAS hydrogel conductivity with water content. The exponential decay model presented in Equation (4) provides a practical empirical equation for the thermal conductivity of PAAS hydrogel within the tested parameter range.

It is worth noting that Equation (4) is applicable to PAAS hydrogel with water contents in the range of 4 ≤ *n* ≤ 260 and at temperatures not exceeding 90 °C. Its validity under other convective conditions or at higher temperatures requires further verification.

## 3. Specific Heat Capacity Model

### 3.1. Experimental Setup

**Figure 4:**
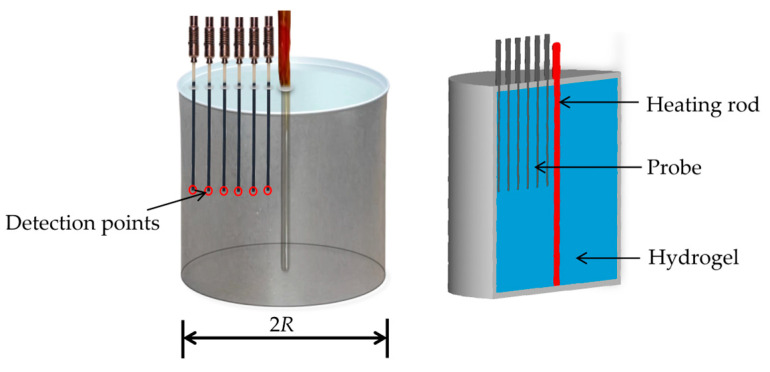
Illustration of the experimental setup for specific heat capacity measurements.

According to the first law of thermodynamics, the heat absorbed by a system is directly related to changes in its temperature and mass [33]. To evaluate the heat absorption capacity of PAAS hydrogels, a dedicated experimental setup was designed and constructed for measuring specific heat capacity based on the calorimetry method, as shown in Figure 4. The setup consists of a heating rod, an insulated container with the radius *R* = 32 mm and the height *h* = 62 mm, and multiple thermocouples uniformly distributed to capture temperature variations. The entire system is thermally isolated from the external environment to minimize heat exchange, thereby ensuring precise and reliable measurements under controlled conditions.

The specific heat capacity *C_n_* is calculated by

(5) $$C_{n}=\frac{P_{2}\;t}{2\pi \;\rho \;h\int_{0}^{R}\left(T\left(r\right)-T_{0}\right)r\;dr},$$

where *P*_2_ is the heating power, *t* is the heating time, *R* is the radial distance from the center of the circular cross-section, and *h* is the sample heigh. Under the initial temperature of the hydrogel *T*_0_ = 20 °C, this experimental setup with the heating power *P*_2_ = 6.58 W and the heating time *t* = 300 s, *T*(*r*) represents the temperature distribution at the end of heating, which is expressed by captured temperature variations by multiple thermocouples uniformly distributed.

Using both differential scanning calorimetry method [26] and the experimental setup of calorimetry method in Figure 4, the specific heat capacities of PAAS hydrogels were measured and summarized in Table 2, where *C_p_* is the specific heat capacity of PAAS powder, *C_w_* is that of water, and *C*_30_ corresponds to a hydrogel with water content *n* = 30.

Differential scanning calorimeter measurements are highly sensitive to local material variations, often resulting in considerable data dispersion. For example, thermal conductivity measurements on a single PAAS hydrogel batch via differential scanning calorimeter exhibited an RSD of approximately 9% across multiple sampling points, with the averaged results of 10 tests summarized in Table 2. In contrast, the custom experimental setup illustrated in Figure 4, designed for larger sample volumes (~154 cm^3^), reduces the influence of local inhomogeneities. Measurements on the same batch yielded a lower RSD of less than 4%, with the average results of five tests also presented in Table 2.

The thermal conductivity results obtained using the custom setup are in good agreement with those from differential scanning calorimetry, while demonstrating greater stability and reliability. Additionally, this setup offers broad applicability, enabling measurements for liquids, powders, and fluid–structure coupling systems, highlighting its versatility.

### 3.2. Experimental Results

The temperature distribution is different for hydrogel with different water content. Through the initial and final temperatures of the hydrogel samples, together with the input heat flow, the specific heat capacity is determined and expressed as a function of water content. The measurement accuracy was within ±3%. At room temperature (20 °C), five independent replications were performed for each condition to ensure consistency and reliability of the results.

**Figure 5:**
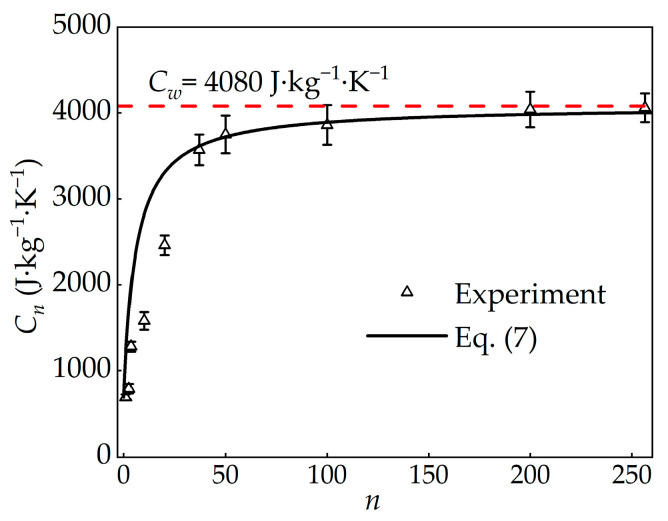
Specific heat capacity of PAAS hydrogels under different water contents.

As shown in Figure 5, with increasing *n*, the PAAS hydrogel network gradually swells, as illustrated in Figure 1. The specific heat capacity of PAAS hydrogels increases exponentially with water content during the absorption and swelling process. At low water contents, the increase is sharp; however, once *n* reaches ~40 or higher, the structural wall of network could be ignored, the rate of increase slows, and the specific heat capacity gradually approaches that of pure water.

### 3.3. Prediction Model

To describe the observed dependence of specific heat capability on water content, the Reuss model for composite materials was adopted [34]. It is expressed as

(6) $$\frac{1}{C}=\frac{V_{f}}{C_{w}}+\frac{1-V_{f}}{C_{p}},$$

where *V_f_* is the volume fraction of water.

Using the measured values of C*_p_* and *C_w_* (Table 2), the specific heat capacity of PAAS hydrogels is expressed in terms of water content *n* as

(7) $$\frac{1}{C_{n}}=\frac{n}{n+1}\frac{1}{C_{w}}+\frac{1}{n+1}\frac{1}{C_{p}}.$$

As shown in Figure 5, the specific heat capacity predicted by Equation (7) is agreement across the full range of water contents. This validates the model’s applicability for describing the specific heat capacity of PAAS hydrogels. Furthermore, the results confirm that as *n* increases, the specific heat capacity asymptotically approaches that of pure water (~4080 J·kg^−1^·K^−1^).

The Reuss model for composite materials, given in Equation (7), provides a robust empirical description of the specific heat capacity of PAAS hydrogels across the full hydration range studied. It is worth noting that, however, all experimental validations were conducted on bulk disc-shaped sample, representing a macroscopic hydrogel rather than thin films or fibers. Additionally, the measurements were performed at temperatures not exceeding 90 °C.

## 4. Thermal Relaxation

The thermal relaxation characteristics of a material are key parameters for evaluating its thermal responsiveness, which describes the dynamic transition from an initial non-equilibrium state to thermal equilibrium. Based on the heat conduction equation, thermal relaxation is commonly characterized by the thermal diffusivity, expressed by

(8) $$\frac{𝜕T}{𝜕t}=\alpha_{n}\nabla^{2}T,$$

where *α_n_* is the thermal diffusion coefficient of the hydrogel under different water content, defined as [35]

(9) $$\alpha_{n}=\frac{k_{n}}{\rho C_{n}}$$

Because both thermal conductivity and specific heat capacity vary nonlinearly with water content (Equations (4) and (7)), the thermal diffusivity of PAAS hydrogel likewise exhibits a nonlinear dependence on water content.

**Figure 6:**
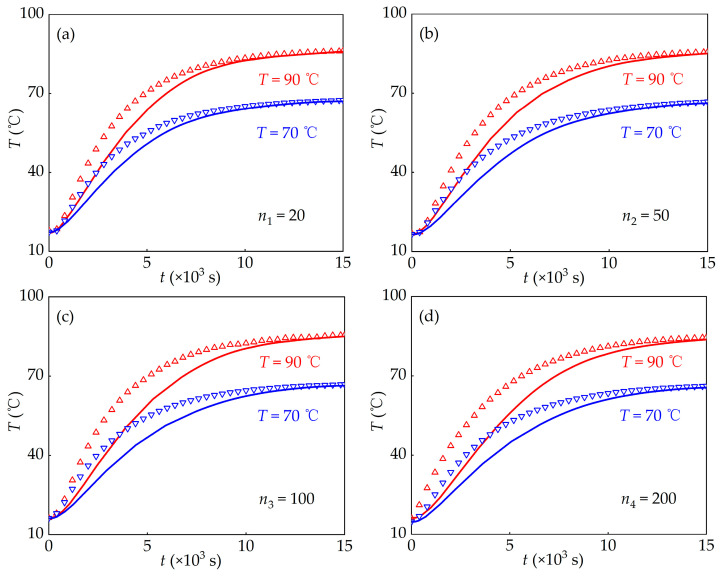
Comparison of thermal relaxation of four samples between the numerical simulation (lines) and experiments (triangles) under 70 °C and 90 °C. (**a**) *n*_1_ = 20; (**b**) *n*_2_ = 50; (**c**) *n*_3_ = 100; (**d**) *n*_4_ = 200.

To examine this behavior, cylindrical copper containers were employed as experimental setup with the diameter 42 mm and height 80 mm. A thermocouple positioned at the center measured the temperature evolution over time. For each test, the container was filled with 110 g of hydrogel. Four PAAS hydrogel samples with different water contents were tested and labeled as *n*_1_ = 20, *n*_2_ = 50, *n*_3_ = 100, and *n*_4_ = 200. The initial temperature was *T*_0_ = 18 °C, the container was kept in a constant-temperature environment, separately 70 °C and 90 °C. The time-dependent temperature variations at the container center are shown using the dots in Figure 6.

Using the thermal conductivity and specific heat capacity of PAAS hydrogel obtained from the experiments (Figure 3 and Figure 5), the heat conduction equation (Equation (8)) was implemented in COMSOL Multiphysics (version 5.3a) to simulate the experimental process. The simulated temperature evolution at the sample center is also shown in Figure 6. Overall, the simulations show good agreement with the experimental trends across all four samples, the deviations between experimental data and simulation results all remained within a reasonable range (<15%). Especially during the later stages of thermal relaxation, there is basically no difference.

However, the discrepancy between numerical and experimental data cannot be neglected, especially for hydrogels with high water content. For the low water-content samples (*n*_1_ = 20 and *n*_2_ = 50), the simulated temperatures were slightly lower than those of the experimental values. As water content increased (*n*_3_ = 100 and *n*_4_ = 200), this difference increased. As the heating experiment was prolonged, the difference gradually decreased between the experimental results and the numerical results based on Fourier heat conduction equation. Meanwhile, for the different temperature environment, the higher the temperature, the greater the difference. Therefore, when PAAS hydrogels are used in thermal protection devices, designs based solely on Fourier’s heat conduction theory may underestimate the actual thermal risk, resulting in higher effective safety coefficients than needed.

This behavior is likely related to a lower degree of cross-linking within the hydrogel network at higher water contents, which reduces interfacial thermal resistance and alters the macroscopic thermal response. Additionally, upon water adsorption, PAAS powder swells (Figure 1), and microstructural heterogeneities during this process cause uneven swelling across different regions, leading to variations in local thermal transport properties. Such localized differences can contribute to non-Fourier heat conduction behavior in macroscopic experiments. In other words, the spatial heterogeneity of the PAAS hydrogel microstructure [36] may give rise to apparent deviations from Fourier conduction, even though numerical simulations are still based on Fourier’s heat conduction theory.

To fully understand the relationship between macroscopic thermal behavior and microscopic mechanisms, a cross-scale thermal framework is required. Future studies should therefore incorporate non-Fourier heat conduction models, which may provide a more accurate explanation of the observed behavior.

## 5. Conclusions

In this paper, we have designed and constructed experimental setups to investigate the heat transfer behavior of PAAS hydrogels. The main findings can be summarized as follows:

(1) The experimental setups successfully measured the thermal conductivity and specific heat capacity of hydrogels with varying water contents, without the need to separately consider liquid, powder, and fluid-solid coupling forms.
(2) Predictive models for thermal conductivity and specific heat capacity were established for PAAS hydrogels within the saturation state under 90 °C. Thermal conductivity increases initially with water content and then decreases, whereas specific heat capacity follows an exponential growth trend.
(3) Thermal diffusivity shows a nonlinear dependence on water content. By adjusting water content, the thermal conductivity can be enhanced by up to ~66% at water content *n* = 30 compared with PAAS powder.

The proposed experimental approach and predictive models provide a valuable framework for regulating the thermal performance of PAAS hydrogels in a fully saturated state. These findings offer practical guidance for the design of thermally efficient hydrogels in heat dissipation and related applications.

## Figures and Tables

**Table 1:** Comparison of thermal conductivity results (W·m^−1^·K^−1^).

Test Materials	Test Methods		Deviation
	Guarded Hot Plate	Transient Plane Source [25]	
PAAS powder *k_p_*	0.56	0.54	3.70%
PAAS hydrogel *k*_30_	0.93	0.95	2.11%
Water *k_w_*	0.61	0.59	3.39%

**Table 2:** Comparison of specific heat capacity test results (J·kg^−1^·K^−1^).

Test Materials	Test Methods		Deviation
	Calorimetry	Differential Scanning Calorimetry [26]	
PAAS powder *C_p_*	690	670	2.99%
PAAS hydrogel *C*_30_	3570	3830	6.79%
Water *C_w_*	4080	4120	0.97%

## Data Availability

The original contributions presented in this study are included in the article. Further inquiries can be directed to the corresponding author.
